# A novel strategy for developing vaccine candidate against Jaagsiekte sheep retrovirus from the envelope and gag proteins: an in-silico approach

**DOI:** 10.1186/s12917-022-03431-0

**Published:** 2022-09-10

**Authors:** Nuha Amin Mahmoud, Abdelmajeed M. Elshafei, Yassir A. Almofti

**Affiliations:** 1 Department of Biochemistry, Genetics and Molecular Biology/ Faculty of Medicine and Surgery, National University, Khartoum, Sudan; 2 Department of Molecular Biology and Bioinformatics, College of Veterinary Medicine, University of Bahri, Khartoum, Sudan

**Keywords:** OPA, T lymphocytes, B lymphocytes, Immunoinformatics, In silico vaccine

## Abstract

**Background:**

Sheep pulmonary adenocarcinoma (OPA) is a contagious lung cancer of sheep caused by the Jaagsiekte retrovirus (JSRV). OPA typically has a serious economic impact worldwide. A vaccine has yet to be developed, even though the disease has been globally spread, along with its complications. This study aimed to construct an effective multi-epitopes vaccine against JSRV eliciting B and T lymphocytes using immunoinformatics tools.

**Results:**

The designed vaccine was composed of 499 amino acids. Before the vaccine was computationally validated, all critical parameters were taken into consideration; including antigenicity, allergenicity, toxicity, and stability. The physiochemical properties of the vaccine displayed an isoelectric point of 9.88. According to the Instability Index (II), the vaccine was stable at 28.28. The vaccine scored 56.51 on the aliphatic index and -0.731 on the GRAVY, indicating that the vaccine was hydrophilic. The RaptorX server was used to predict the vaccine's tertiary structure, the GalaxyWEB server refined the structure, and the Ramachandran plot and the ProSA-web server validated the vaccine's tertiary structure. Protein-sol and the SOLPro servers showed the solubility of the vaccine. Moreover, the high mobile regions in the vaccine’s structure were reduced and the vaccine’s stability was improved by disulfide engineering. Also, the vaccine construct was docked with an ovine MHC-1 allele and showed efficient binding energy. Immune simulation remarkably showed high levels of immunoglobulins, T lymphocytes, and INF-γ secretions. The molecular dynamic simulation provided the stability of the constructed vaccine. Finally, the vaccine was back-transcribed into a DNA sequence and cloned into a pET-30a ( +) vector to affirm the potency of translation and microbial expression.

**Conclusion:**

A novel multi-epitopes vaccine construct against JSRV, was formed from B and T lymphocytes epitopes, and was produced with potential protection. This study might help in controlling and eradicating OPA.

**Supplementary Information:**

The online version contains supplementary material available at 10.1186/s12917-022-03431-0.

## Background

Ovine pulmonary adenocarcinoma (OPA) (syn: sheep pulmonary adenomatosis) is a naturally occurring lung cancer, also known as Jaagsiekte “driving sickness” in Africa [1]. It is a transmissible, neoplastic disease affecting the lungs of sheep, and, rarely of goats. It is caused by Jaagsiekte sheep retrovirus (JSRV) [2–5]. The disease first appeared in South Africa in 1915. Nowadays, it has spread throughout the world mainly in sheep-raising countries like Europe, Africa, Asia, and America. The impact of this disease on the agricultural industry is important on an economic and welfare level [1, 6–8]. OPA accounts for nearly 70% of all sheep tumors since it primarily targets domestic sheep (Ovis aries). Nevertheless, a few cases of Sardinian moufflon (Ovis musimon, a species of wild sheep) and domesticated goats have been reported [6–8].

Concerning the family Retroviridae, the JSRV is a member of the beta retrovirus genus. JSRV resembles a simple retrovirus. This beta retrovirus is the only known virus capable of instigating the formation of naturally occurring lung adenocarcinomas [6–11]. In alveolar and bronchial secretory epithelial cells, JSRV stimulates neoplastic transformation [12, 13]. Eventually, the majority of the lung is occupied by tumors. Fluid overproduction in the lung is frequently seen with tumor growth, impairing normal respiration even further [4, 12–14].

As the tumor growth progresses, it is accompanied by the secretion of copious lung fluid that contains an infectious virus, which may be a prominent feature of OPA. Affected sheep become increasingly tachypneic at rest with an amplified abdominal component to their breathing [14]. It may take several months to several years for this disease to incubate [15, 16]. Infected sheep, including sub-clinically infected sheep, can spread OPA through aerosols and droplets inhaled through the respiratory route [13]. Sheep of all ages are susceptible to horizontal transmission, but young lambs are especially susceptible. Close contact is believed to extend transmission [13, 17]. JSRV also occurs in milk and colostrum, which might transmit the virus to nursing animals [18, 19]. JSRV-infected sheep may not exhibit any clinical manifestations during their lifetime. As a result, OPA can be spread into new flocks by interacting with infected but asymptomatic normal animals [12].

OPA is clinically, radiologically, and histologically similar to bronchioloalveolar cancer (BAC), a form of human cancer. Due to these similarities, researchers have hypothesized that the JSRV or its related virus might be involved in human cancer [7, 14, 20]. Since OPA and human lung tumors are histologically similar, OPA is considered a natural animal model for adenocarcinomas of mixed subtypes in humans. There were multiple reports of a retroviral etiology for these human tumors, and there have been cases where antigens within these tumors were similar to betaretroviral Gag proteins [3, 6–8, 14, 21].

The genus retrovirus is an RNA virus that infects vertebrate species and various non-vertebrate species [7, 22]. The virus envelope is spherical with spikes of virus-encoded glycoproteins, with an outer diameter of 80–100 nm. Viral proteins and host-cell membrane elements (proteins and lipid bi-layer) are enclosed in this envelope. The viral genomes consist of two copies of linear, single-stranded RNA. An infectious virus contains a genome of 7.58 kb, organized as 5’- gag- pro- pol- env – 3’, which encodes all its proteins [23, 24]. Several structural proteins encoded by the gag gene encapsulate the viral RNA genome and form the core of the viral particle; these include nucleocapsid, matrix, and capsid [13, 23]. The pol gene is situated adjacent to or overlaps the gag gene, and encodes enzymes that are essential for viral life, which include reverse transcriptase and integrase. In viral budding and assembly, the viral pro gene encodes the viral protease that matures the viral particle [14, 23]. The env gene encodes both the surface and transmembrane domains of the envelope protein that is embedded in the membrane; during infection, the Env protein binds the cell receptor [10, 11, 24].

OPA was first pronounced in the early nineteenth century. It is a chronic wasting illness, usually considered a progressive respiratory distress disease resulting in emaciation. However, it may also be predisposing to secondary bacterial pneumonia, causing sudden death even after antibiotic treatment. In many countries raising sheep, OPA is a significant disease with a cost-valuable influence especially, upon first exposure to the virus where up to 80% of the flock would be lost. While continuing losses might be as high as 20% per annum in some flocks [24]. In preclinical forms, the disease could not be reliably diagnosed. Thus it is difficult to exclude the disease from the flocks [23–25]. Despite the use of high sensitive assays, such as immunoblotting or enzyme-linked immunosorbent assays, sheep infected with JSRV would not produce detectable antibodies against the virus, eliminating serological tests for diagnosis of infection [16, 26, 27]. Successful control and promising disease eradication have been hampered by the lack of suitable diagnostic tests, treatments, or vaccines. So it remains an essential problem in most countries where sheep are farmed. Thus, this study aimed to design a multi-epitopes vaccine against OPA using structural proteins of JSRV.

## Results

### OPA structural proteins retrieval and sequences alignment

**Fig. 1:**
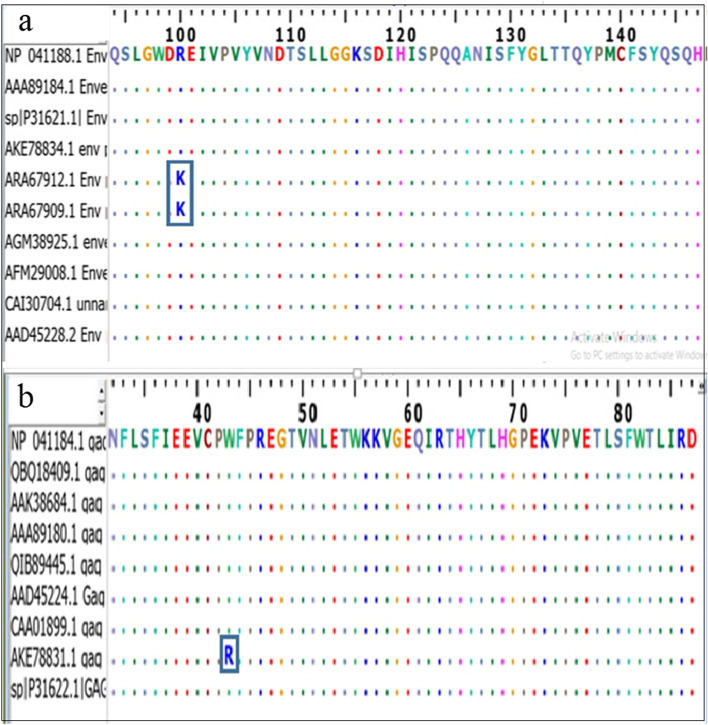
A multi-sequence alignment (MSA) was performed using the BioEdit software and the ClustalW on the strain retrieved forms. **a** The envelope protein and **b** The gag protein. Within the rectangle, the letters indicated the non-conserved areas, and the dots indicated the conserved regions

Based on the alignment of all retrieved strains of both the envelope and gag proteins with the ClustalW tool, which is available in Bioedit software. Only the 100% conserved epitopes were chosen (Fig. 1). The conserved regions were found by the identity of the amino acid sequences amongst the retrieved sequences. In contrast to non-conserved epitopes, both B and T lymphocytes epitopes with 100% conservancy were included for further analysis.

### Prediction of B-cell epitopes

ABCpred tool received reference sequences of the envelope and gag proteins for B cell epitopes prediction. The predicted B cell epitopes were graded according to their score, by a trained recurrent neural network. The greater score of the peptide indicated the greater probability to be a B cell epitope passing a threshold of 0.51. Moreover, the epitopes were assessed for their conservancy, antigenicity, allergenicity, and toxicity. Based on these assessments, the results showed that there were 25 and 14 peptides from the envelope and gag proteins that were predicted as B cell epitopes, respectively. Afterward, epitopes were sorted and ordered in descending manner based on their high antigenicity scores. B cell epitopes elected for vaccine construction from the envelope and gag proteins were based on the antigenicity scores equal to or greater than 1.250 and 0.800, respectively. Accordingly, eight epitopes from each protein were entered into the vaccine construction (Table 1). The rest of the predicted epitopes were shown as supplementary files (S1).

**Table 1:** A list of the eight predicted B cell epitopes from the envelope and gag proteins. The antigenicity, allergenicity, and toxicity of epitopes were assessed

Protein	Predicted epitopes	Start	APCPred prediction score	Vaxijen^a^ antigenicity
**Envelope protein**	GTKYGDVGVTGF^b^	278	0.61	1.7111
	HISPQQANISFY^b^	120	0.66	1.6667
	EQVQSINFRMKI^b^	451	0.64	1.6045
	QYPMCFSYQSQH^b^	136	0.62	1.402
	GLTTQYPMCFSY^b^	132	0.6	1.3312
	ISGIDEKTGKKS^b^	164	0.68	1.2867
	HLSIGIGIDTPW^b^	191	0.65	1.2565
	YPRVTISGIDEK^b^	159	0.59	1.2504
**Gag protein**	TDTQLNFLPGAY^b^	361	0.65	1.3888
	LNFLPGAYAQIS^b^	365	0.73	1.2633
	QEKGALTSKDEL^b^	165	0.84	1.1569
	PPPPPSLKMHPS^b^	123	0.6	1.103
	DWKQTARACLSG^b^	308	0.61	1.0534
	FKQLKELKIACS^b^	272	0.54	0.8964
	NRQQGIQTSYEM^b^	340	0.52	0.8518
	SSTKTEDLSKVR^b^	389	0.65	0.824

^a^The threshold for the Vaxijen antigenicity was 0.4

^b^Represents the epitopes inter in the structure of the vaccine construct. All the predicted epitopes were shown to be non-allergen and non-toxic

### Prediction of cytotoxic T lymphocyte epitopes

Reference sequences of the envelope and gag proteins were investigated to predict epitopes interacting with the MHC-I ovine allele; using the IEDB MHC-1 binding prediction tool. IEDB creates prediction probabilities from an Artificial Neural Network (ANN) with half-maximal inhibitory concentration (IC50) ≤ 100. There were 195 and 72 predicted epitopes from envelope and gag proteins, respectively, predicted interacting with different MHC-1 alleles. The predicted epitopes were subjected to conservancy using BioEdit software. Furthermore, the antigenicity, allergenicity, and toxicity were also examined for each predicted epitope. Based on these assessments, there were 12 and 36 epitopes from the envelope and gag proteins that were predicted as cytotoxic T cell epitopes, respectively. These epitopes were ranked according to their highest antigenicity scores (equal to or greater than 0.600 and 1.050 for the envelope and gag proteins, respectively). Thus the first eight epitopes with the highest-ranking score of antigenicity from each protein were chosen to form chimeric multi-epitopes JSRV vaccine alongside the other parameters (Table 2). The rest of the predicted epitopes were shown as supplementary files (S2).

**Table 2:** The eight predicted cytotoxic T cells epitopes, their antigenicity, allergenicity, and toxicity from the envelope and gag proteins

**Protein**	**Epitope**	**Alleles**	**Start**	**End**	**Vaxijen**^a^ **Antigenicity**
**Envelope protein**	YKWICVTKK^b^	BoLA-T2C	468	476	1.7367
	HISPQQANI^b^	BoLA-T2C	120	128	1.5736
	ISFYGLTTQ^b^	BoLA-T2a	128	136	1.1064
	RGVAKGEQV^b^	BoLA-HD6	330	338	1.0778
	GVTGFLYPR^b^	BoLA-T2a	285	293	0.9625
	MKIQCHANY^b^	BoLA-D18.4	460	468	0.8693
	SLLGGKSDI^b^	BoLA-T2C	111	119	0.7804
	HLNCSNCIL^b^	BoLA-T2C	317	325	0.6677
**Gag protein**	TSYEMLIGE^b^	BoLA-T2a	347	355	1.5370
	CFKNLTIAL^b^	BoLA-T2C	179	187	1.4302
	RKKGDLSDF^b^	BoLA-D18.4	452	460	1.3402
	CLDFDNDEL^b^	BoLA-T2C	88	96	1.1209
	GQPGHRAAV^b^	BoLA-D18.4	513	521	1.0927
	RQAQRLGEV^b^	BoLA-D18.4	240	248	1.0927
	NSGCFVCGQ^b^	BoLA-T2a	506	514	1.0792
	AMAAALQGK^b^	BoLA-T2a	476	484	1.0711

^a^The threshold for the Vaxijen antigenicity was 0.4

^b^Represents the epitopes entered in the structure of the vaccine construct. All the predicted epitopes were shown to be non-allergen and non-toxic

### Vaccine construction

**Fig. 2:**
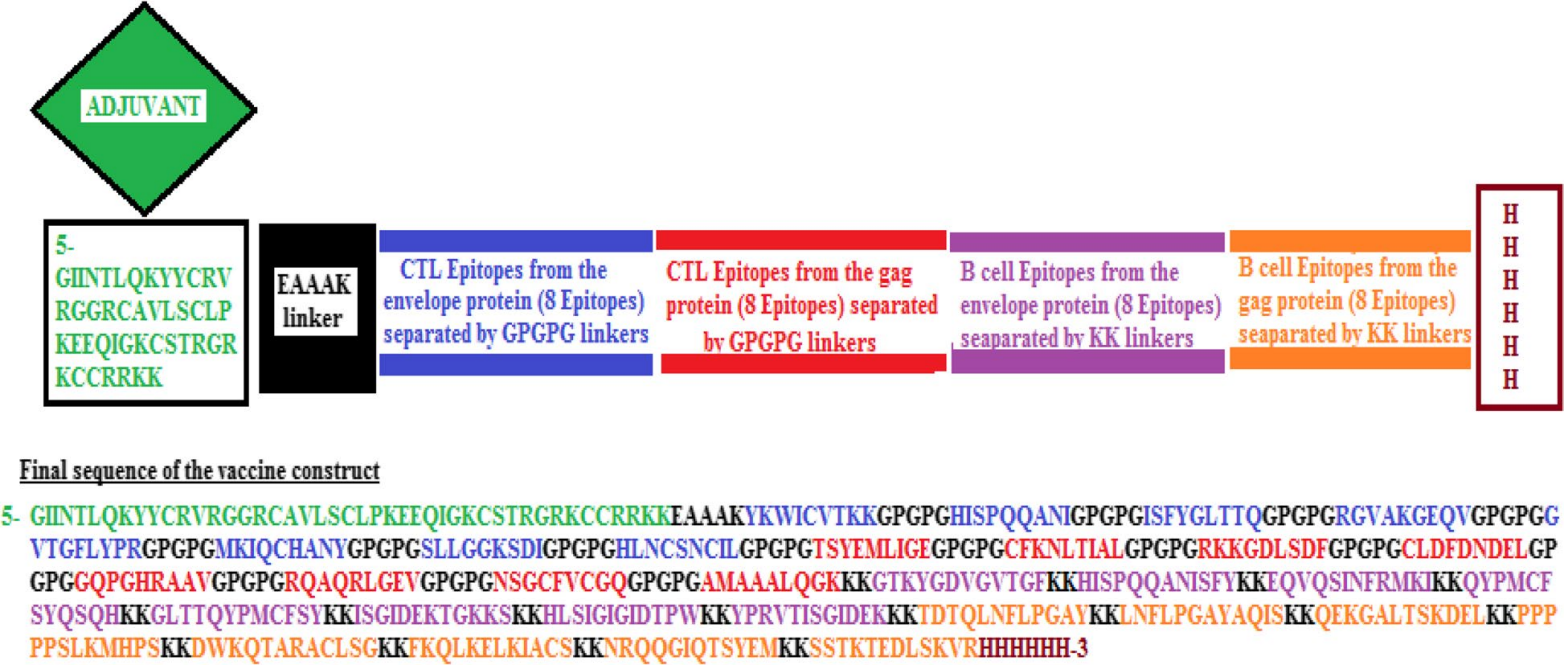
multi-epitope vaccine design. MHC1 epitopes for the envelope protein (blue colour), MHC1 epitopes for the gag protein (red colour), B cell epitopes for the envelope protein (purple colour), and B cells epitopes for the gag protein (orange colour). For both the envelope and the gag proteins, MHC-1 epitopes were linked to the GPGPG linker, while the B cells epitopes for both proteins were linked by the dipeptide KK linkers. As an adjuvant, Humanβ-defensin-3 (green colour) was added to the N terminals and is linked by the short EAAAK linker (black colour). At C-terminal, the his-tag (6 H) was added (brown colour)

To assemble the vaccine construct, 16 linear B-cell epitopes and 16 cytotoxic T-cell epitopes were selected from both the envelope proteins and the gag proteins, along with adjuvants, linkers, and His-tags. The overall amino acids of the vaccine construct were 499 amino acids (Fig. 2). Based on a VaxiJen score of 0.8932, the vaccine construct was shown to be antigenic and it also showed a non-allergic response.

### Physicochemical properties of vaccine construction

According to the ProtParam server, the vaccine construct has a molecular weight of 53.3 kilodaltons. A theoretical isoelectric point value (pI) of 9.88 was calculated. Overall, there were 30 negatively (Asp + Glu) and 82 positively (Arg + Lys), and the charged residues and Cys % was 3.6%. At 280 nm, the extinction coefficient (M − 1 cm − 1) measured in water was 42,955, which assumes that each pair of Cys residues is a cysteine. Similarly, the ProtParam server showed an estimated half-life of 30 h for mammalian reticulocytes (in vitro), > 20 h for yeast (in vivo), and > 10 h for Escherichia coli (in vivo). The instability index (II) of this vaccine was 28.28, indicating the stability of the construct. ProtParam server also calculated the aliphatic index and the GRAVY value for the vaccine and they were 56.51 and -0.731 respectively, thus the vaccine was classified as hydrophilic.

### Structural analysis

#### Vaccine constructs secondary structure

To predict the protein secondary structure, the RaptorX server was applied. The constructed vaccine demonstrated 12% H, 17% E, and 70% C. The solvent access was 70% E, 13% M, and 16% B. 55 (11%) positions were predicted as disordered. For transmembrane helix prediction, a MEMSATSVM from the PSIPRED server was used and revealed one insignificant transmembrane helix with a score of -0.788872. This result was confirmed by the result of the TMHMM server that showed the number of predicted TMHS was zero.

#### Prediction, refinement, and adaptation of the vaccine tertiary structure

**Fig.3:**
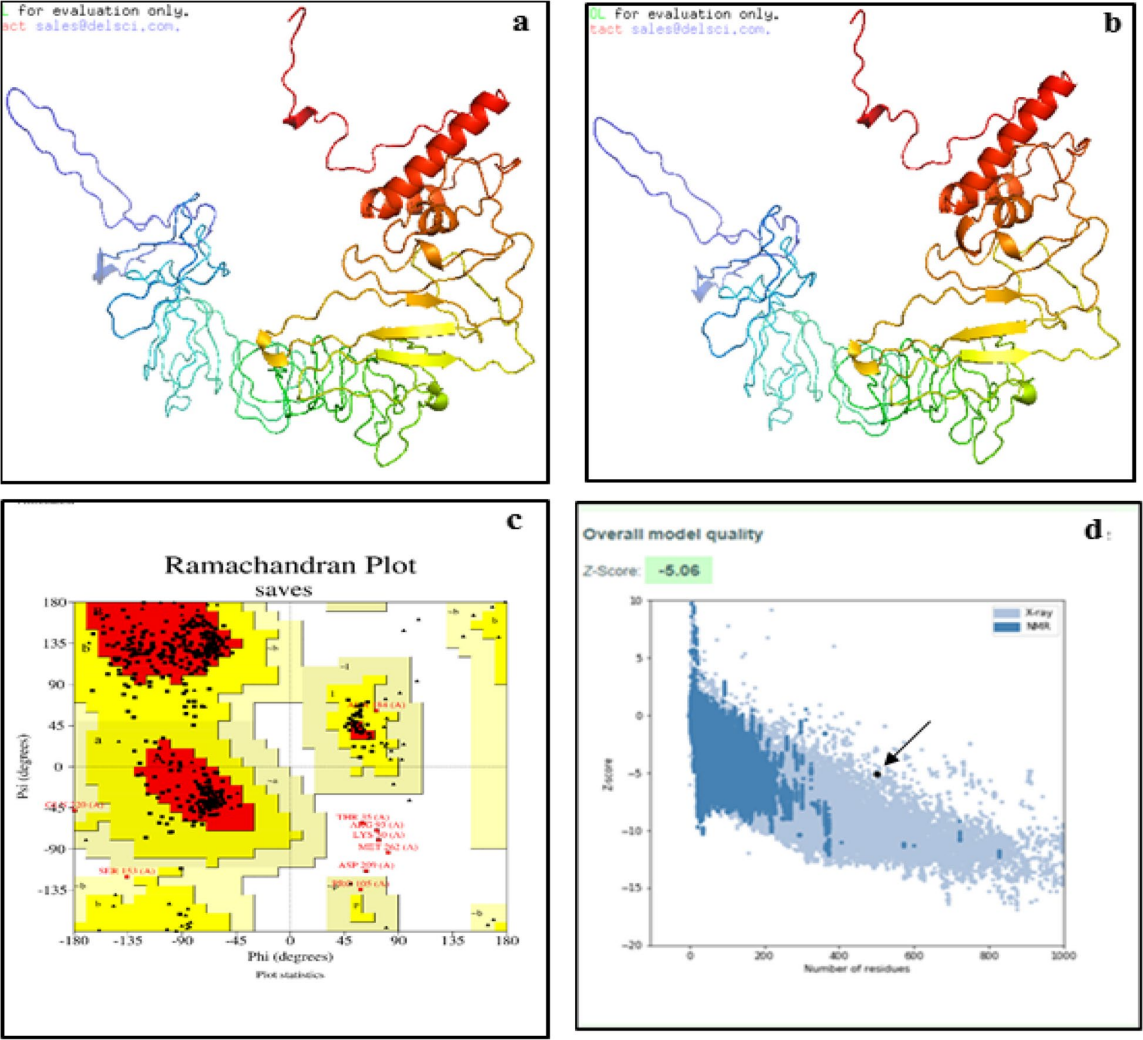
**a** RaptorX’s homology modeling tool was used to create a 3D model of the vaccine construct. **b** The Galaxyrefiner tool refined the 3Dmodel. **c** A Ramachandran plot analysis of the validated, refined model yielded 98.7% residues in the allowed regions. **d** the ProSA-server, giving a Z-score of − 5.0

RaptorX server predicted the tertiary structure of the constructed vaccine with the PDB file models. The best-modeled PDB file of the 3D structure (Fig. 3a) was refined by the Galaxy refiner server, to enhance the global and local structure quality on average (Fig. 3b). The Ramachandran plot was used to identify which torsional angles were permissible and to get insight into the structure of peptides of the refined vaccine PDB file. The result revealed the rate of residues in the allowed regions was 98.7% (Fig. 3c). The refined vaccine construct model was also assessed with the ProSA server for potential errors. The Z-score demonstrated -5.06 indicating the favorable structure of the vaccine (Fig. 3d).

#### The solubility of the vaccine construct

**Fig. 4:**
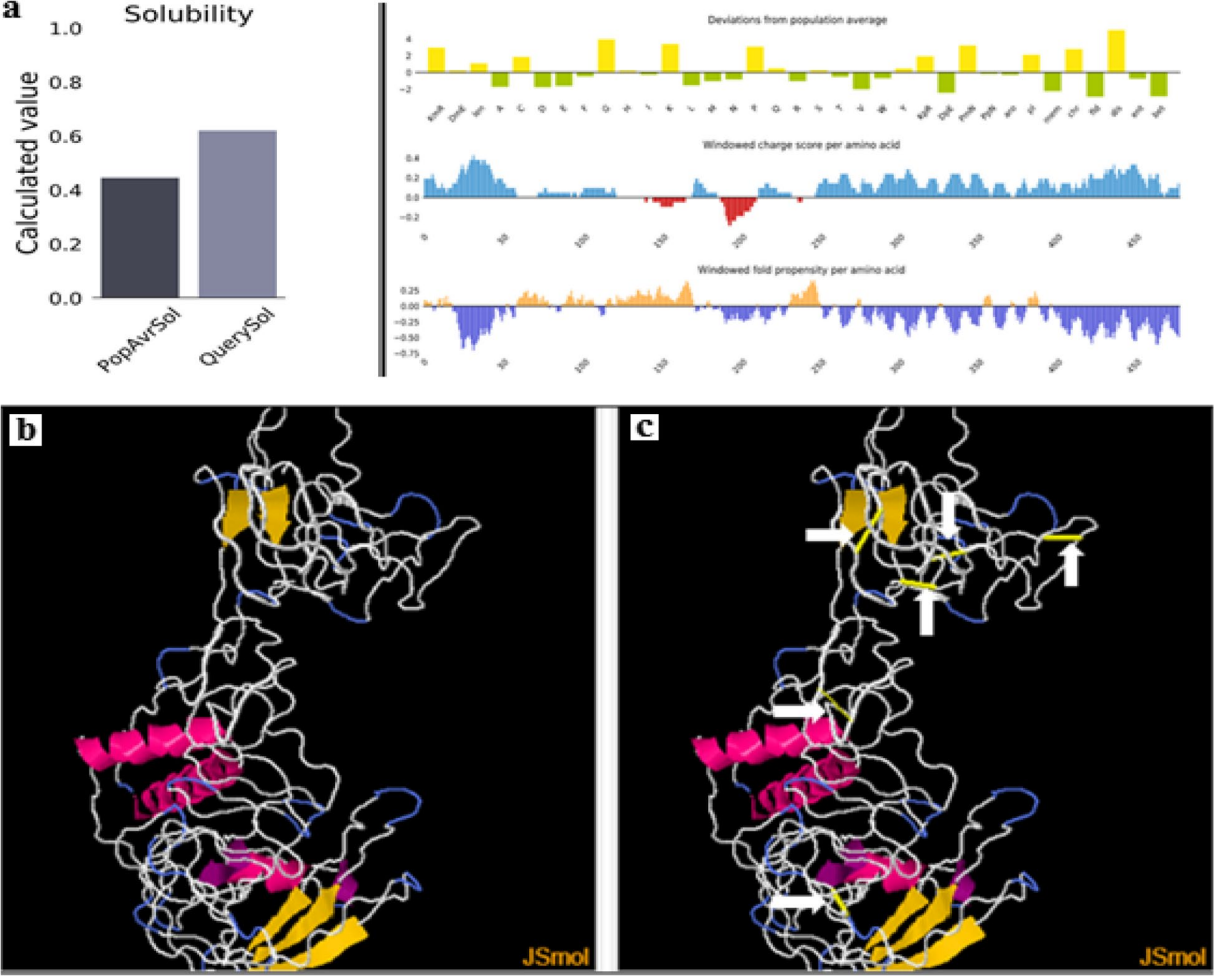
**a** As compared to *E. coli*, the vaccine was soluble, with a predicted scaled solubility of 0.621 versus 0.45 of the *E. coli* solubility based on the population averages. **b** Engineering disulfide bonds to stabilize vaccine constructs showing the wild-type version. **c** The mutant version showed six disulfide bonds added in regions of golden sticky forms indicated by white arrows

The server Protein-Sol was used to determine the scaled solubility of the vaccine construct, which was 0.621 versus 0.45 of the population solubility of *E. coli* (Fig. 4a). SOLpro was then used to confirm the predicted solubility of the vaccine. The proposed vaccine was predicted to be soluble upon overexpression with a probability of 0.819106.

#### Stability of the vaccine via disulfide bonds prediction

To detect the number and location of the disulfide bonds, the DIpro tool server was used. The total number of cysteine residues was 18, and the predicted numbers of bonds were seven bonds. Cysteine residues were predicted to form the disulfide bonds at positions 18–23, 33–40, 41–55, 177–205, 250–253, 318–336, and 448–464. Additionally, mutated residues containing cysteine in the highly mobile region of the protein sequence were used to augment the stability of the vaccine construct. According to Disulfide by Design 2.0 (DbD2) server with optimum Chi3 angle ranging from + 97 to -87 with tolerance 30 and the maximum Ca-Cb-S angle with tolerance114.60, 49 pairs of amino acid residues were likely shown to form disulfide bonds. Among these 49 amino acids residues, only six residues were chosen to be replaced by cysteine based on the B-factor value (ranged 6.950–17.410) and energy value less than 3.0 to form disulfide bonds at these positions 74–98, 124–151,136–156, 246–285, 265–292, and 354–380 (Fig. 4 b-c).

### Immunological analysis

#### Molecular docking of the vaccine construct with MHC Class 1 antigen

**Fig. 5:**
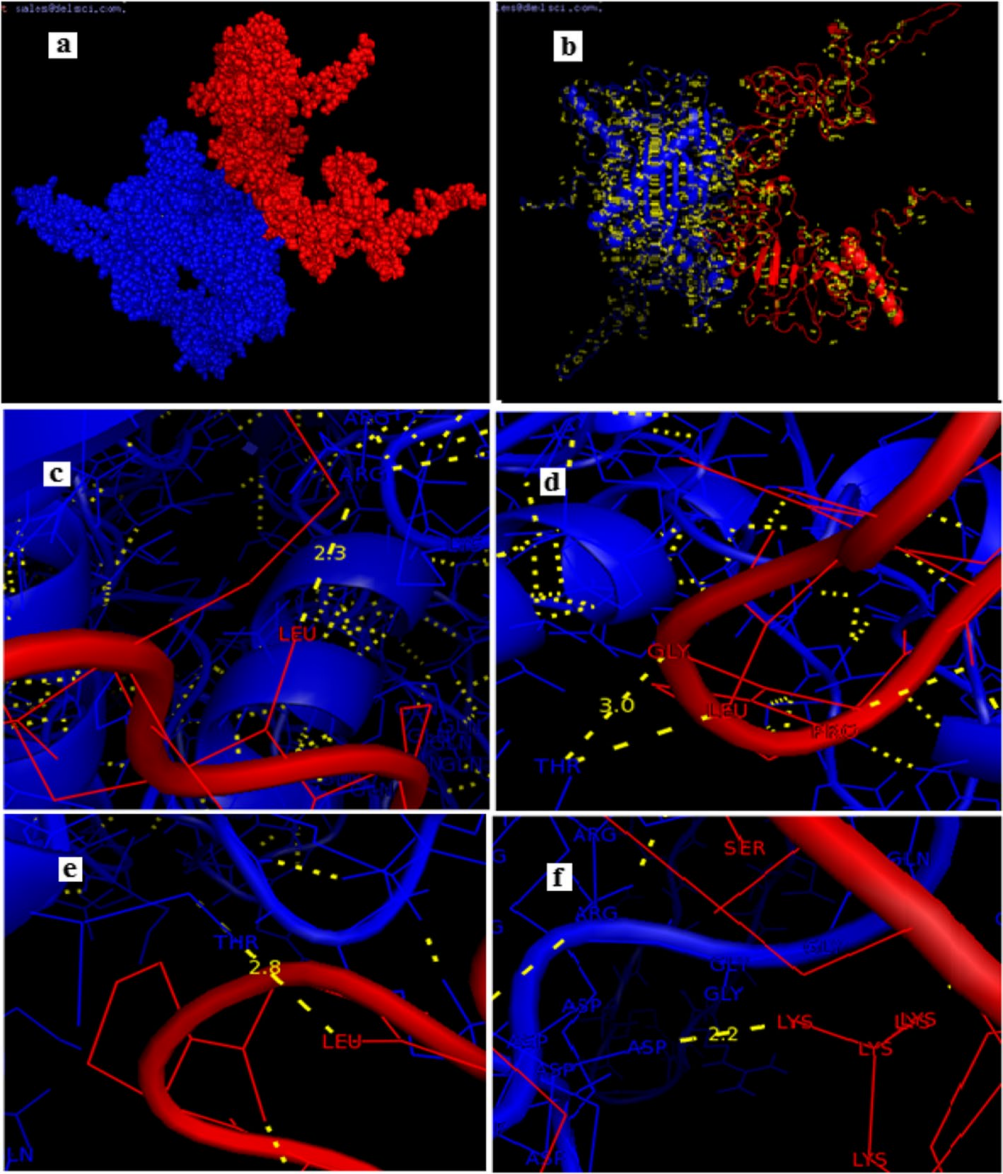
Molecular docking between the vaccine as a ligand (blue colour) and the ovine MHC antigen 1 as a receptor (red colour). **a** A modeled spheres structure of the vaccine docked with MHC antigen 1. **b** A cartoon representation showing hydrogen bonding (yellow dashes) between the ligand and the receptor. **c** Showed 2.3 Å hydrogen bonding between ARG’173 of the ligand to LEU’213 of the receptor. **d** Showed 3.0 Å hydrogen bonding of THR’12 of the ligand to GLY 186 of the receptor. **e** Showed 2.8 Å hydrogen bonding of THR’166 of the ligand to LEU 185 of the receptor. **f** Showed 2.2 Å hydrogen bonding of ASP’358 of the ligand to LYS 271 of the receptor

For the docking analysis; the ClusPro server was used to dock the vaccine construct against the ovine MHC-1 antigen. As shown in (Fig. 5 a-b) the attractive binding energy bound the constructed vaccine to the MHC-1 antigen was -1264.2 kcal/mol. The low (negative) energy points out to a stable system and thus expected strong binding interactions. The model selected had the lowest energy score obtained amongst all 30 predicted docked complexes indicating the highest binding affinity. Figure 5 (c-f) showed the strong interaction between the vaccine and the ovine MHC-1 antigen in terms of hydrogen bonds.

#### Immune simulation

**Fig. 6:**
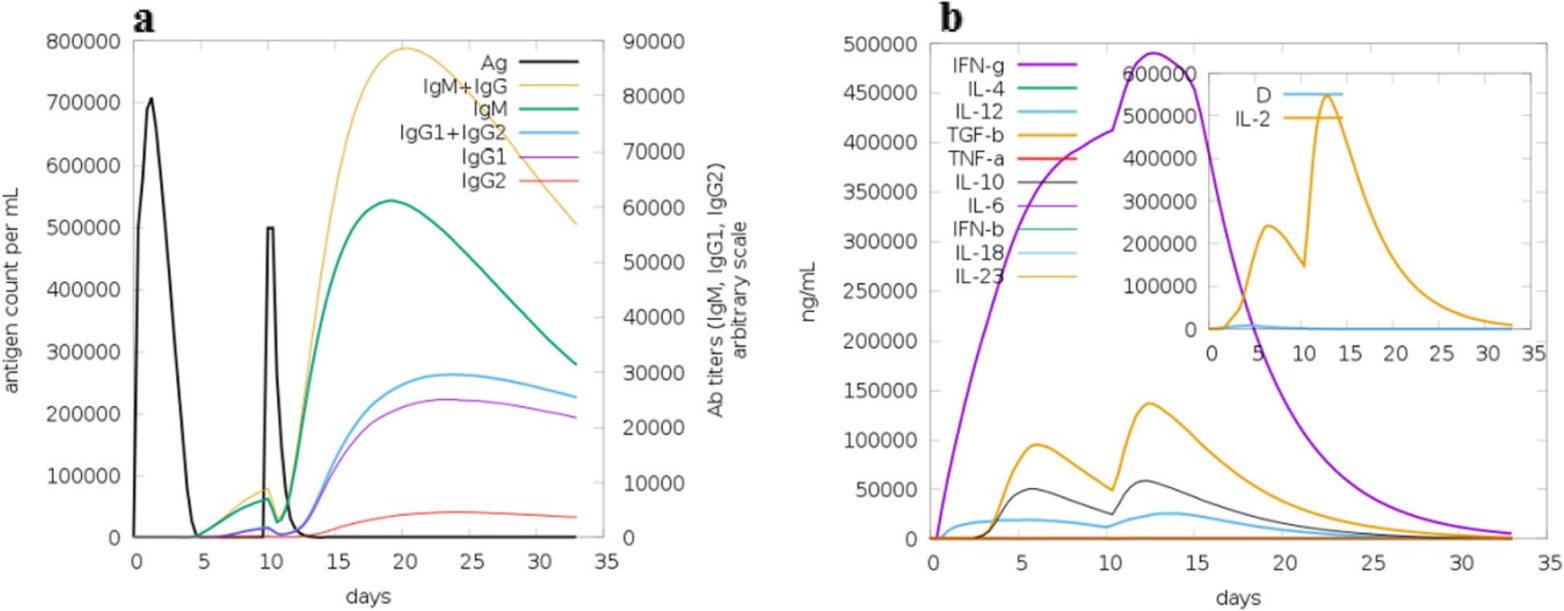
**a** Immunoglobulins production increased in response to exposure to antigen injections with a marked decrease in the antigen concentration. **b** The levels of cytokines induced by the injection of the vaccine. The insert plot demonstrated the danger signal using IL-2 with the Simpson index shown by the dotted line. The smaller the index value, the lower the diversity

**Fig. 7:**
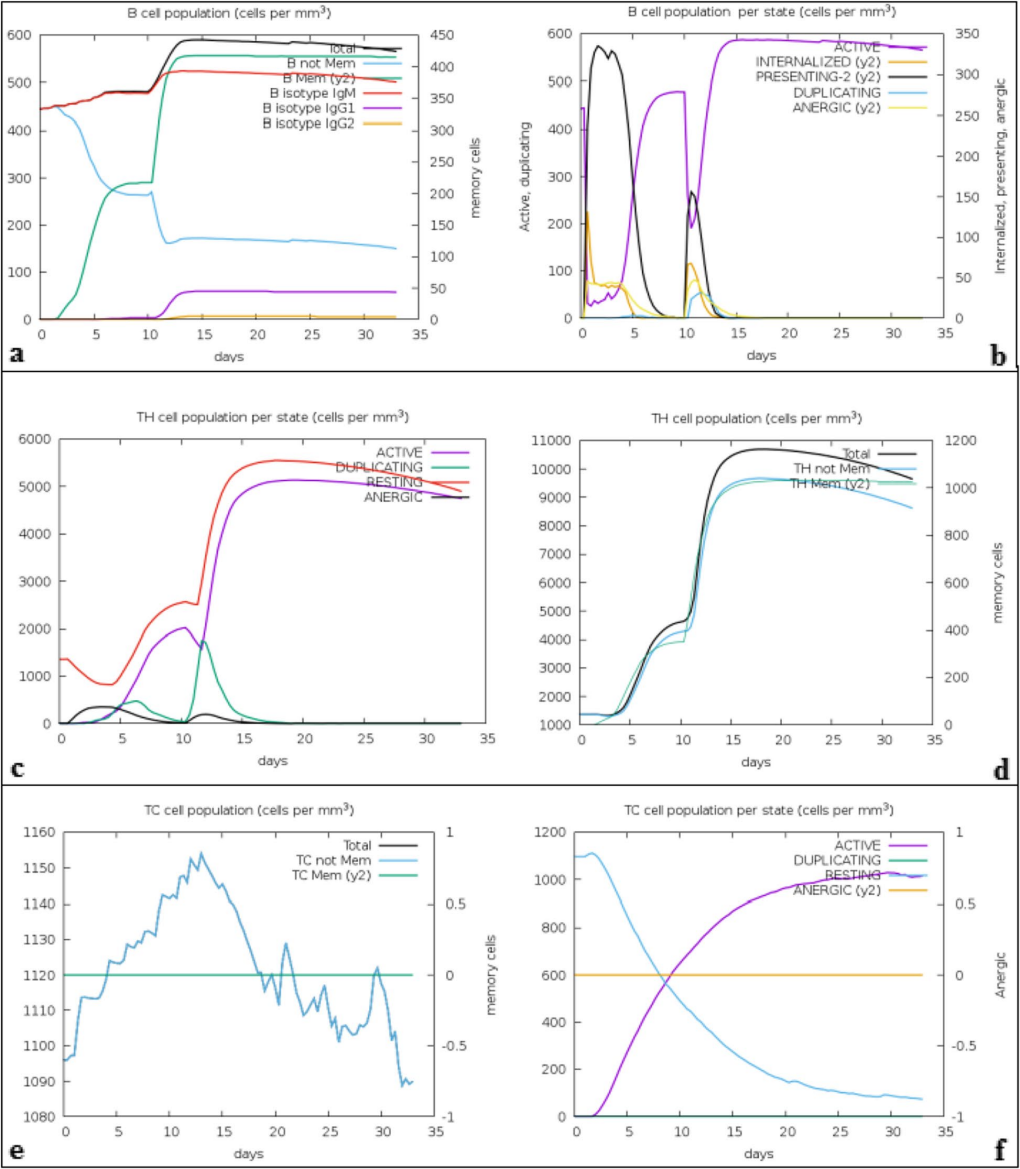
**a** Showed the B-cell populations with a marked increase in the memory and non-memory immunoglobulins. **b** Showed the B-cell population internalized percentages. **c** Showed the increased level in the populations of the active T helpers. **d** Showed the increased level of the active T helpers as memory cells. **e** Showed the increased level in the populations of the active T cytotoxic cells as memory cells, and **f** Showed the resting state (cells not exposed to antigen) and anergic state (tolerance to the antigen exposures) of T cytotoxic cells

C-ImmSim server was exploited to mimic the immune responses produced due to exposure to the vaccine. The first contact between the immune system and the vaccine (antigen) resulted in an elevated level of IgM + IgG accompanied by a marked reduction in the vaccine concentration. The elevation of the level of the antibodies’ production is based on the nature of the antigen and is often produced in low concentrations. (Figure 6-a) showed the remarkably increased concentration of IgM + IgG upon the first injection of the vaccine. Repeated injection of the vaccine resulted in an increased level of IgM, IgG, and the memory cells with a diminished concentration of the vaccine (antigen). This demonstrated that the immunoglobulins had a higher affinity to the vaccine and developed immune memory that benefited the host in subsequent exposure to the virus. For IFN-γ production, exposure to the vaccine showed remarkable production of induced IFN-γ compared to the other cytokines. Simpson index D showed the level of danger as cytokines level increased which may result in complications during the immune response (Fig. 6-b). On the other hand, the T-lymphocytes demonstrated high populations of cell responses accompanied by memory development. Strikingly T cytotoxic and T helper lymphocytes populations remain elevated upon all exposure time (Fig. 7).

#### Molecular dynamic simulation

**Fig. 8:**
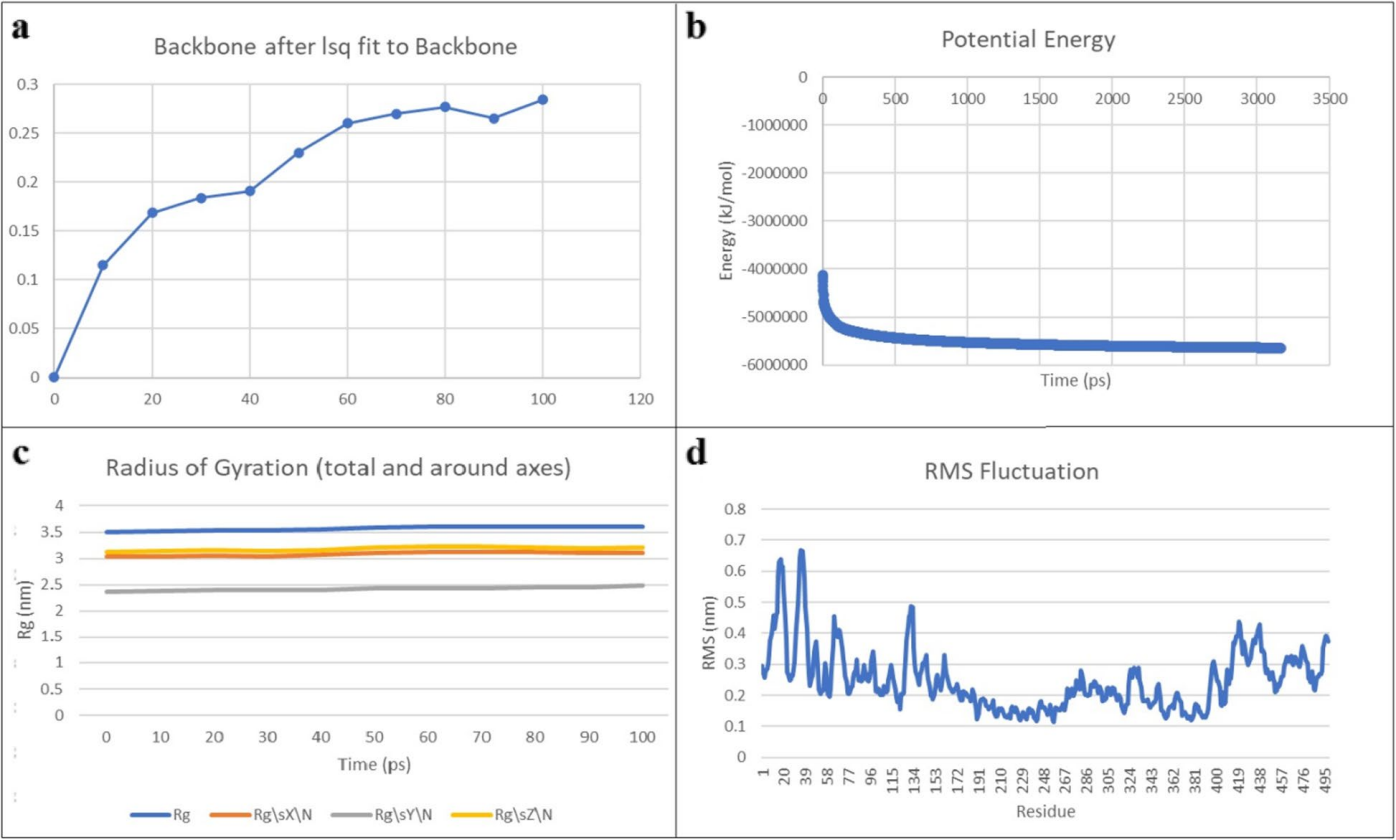
**a** shows the simulation of the backbone of the vaccine structure. **b** The potential energy of the vaccine. **c** the radius of the gyration plot demonstrated the compactness of the vaccine around its axes. **d** RMSF plot of the vaccine construct shows high fluctuations, indicating high flexibility stability

To examine the stability of the vaccine, MDS was performed using the GROMACS algorithm (Fig. 8). The simulation of the vaccine backbone was shown in (Fig. 8-a). The potential energy demonstrated − 5.0e + 06 kJ/mol (Fig. 8-b). Trajectory analysis was conducted attempting to examine the flexibility and the stability of the vaccine. The radius of the gyration plot demonstrated the compactness of the protein around its axes (Fig. 8-c). RMSF plot of the backbone provided the least fluctuations, strengthening the stability of the vaccine over time (Fig. 8-d).

**Fig. 9:**
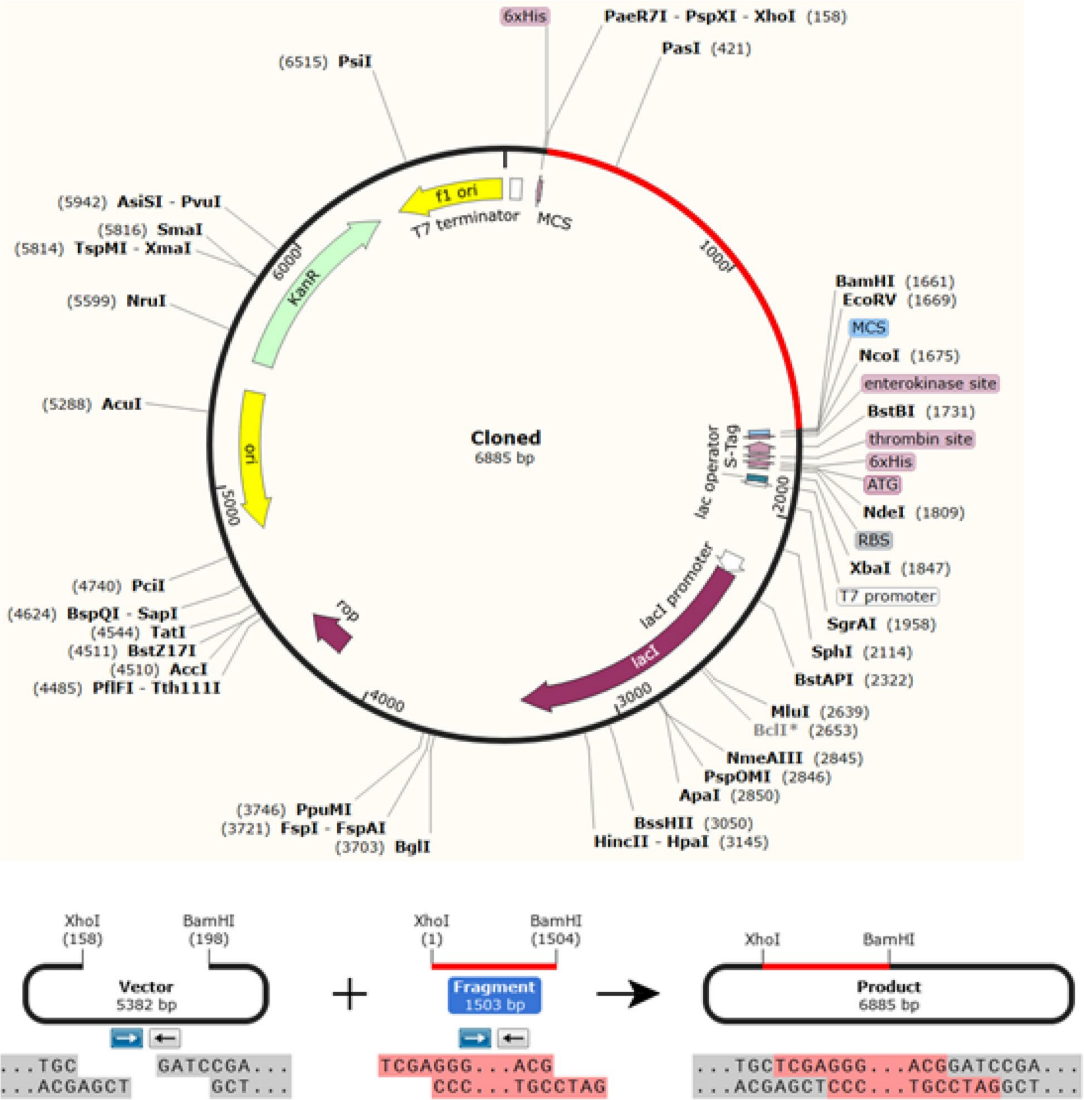
The final vaccine construct sequence cloned into the pET30a ( +) expression vector. The vector is shown in black, and the vaccine DNA sequence is highlighted in red. The DNA sequence is typically inserted in the MCS between the BamH1 and Xho1 cutting sites

#### In silico cloning and codon adaptation

Back-translation of the protein sequence into a DNA sequence generated a codon adaptation index value (CAI-value) of 1.0% for the improved DNA sequence. The GC content of this improved sequence was 52.037 which indicated a satisfactory GC content. (Figure 9) depicted the cloning of the DNA sequence into the pET- 30a ( +) vector using the multiple cloning site (MCS) of the vector, which was located between the BamHI and Xho1 restriction enzymes cutting sites at the C and N vicinities of the vector.

## Discussion

OPA is a contagious, chronic and progressive neoplasm that is caused by the sheep retrovirus, called the Jaagsiekte sheep retrovirus (JSRV) [9]. The disease leads to significant economic and veterinary losses among sheep producers. The virus is uniquely able to transform lung cells such as type II alveolar cells and Clara cells (bronchiolar nonciliated cells). This type of transformation made JSRV unique among retroviruses [14]. The disease has been detected worldwide among various breeds of sheep and rarely goats, with a typical serious influence on infected flocks. Moreover, OPA displayed excellent morphologically and histologically similar characteristics to human lung carcinogenesis [9, 11, 14]. Meanwhile, neither a vaccine nor serological diagnostic approaches to identify OPA were available [7, 28].

In silico immunoinformatic approaches are increasingly used as the first step when developing vaccine candidates, as they are cost-effective and have time-saving benefits. During vaccine development, this approach can also overwhelm limits including genetic variations, antigenic drifts, and antigenic shifts [29]. Recently, epitope-based vaccines researches for different animals are vastly growing with successful results [29–31]. This study is the first in silico analysis study that aimed to predict multi-epitopes vaccine against the Jaagsiekte retrovirus.

The JSRV genome is uniquely characterized by its native envelope structural protein. The active oncogene consists of the cytoplasmic domain of the transmembrane glycoprotein and as well as, other cytoplasmic domains found in the surface glycoprotein [32]. Even though other regions of the envelope protein have also been implicated in transformation, the cytoplasmic tail of the envelope transmembrane protein is a crucial feature [11, 14, 33, 34]. JSRV gag protein is the major structural polyprotein constituent encompassed in intracellular trafficking processes and orchestrates viral assemblies and dis-assemblies. Also, gag protein regulates viral gene expression and mediates correct encapsidation of Pol protein [35]. The viral genome and the accessory proteins were also involved in the Spatio-temporal regulation of the essential, viral, and enzymatic reactions as well as being essential for viral budding [36].

In addition to their role in cell signaling, TMH proteins are essential for membrane-impermeable molecule transport, cell-to-cell communication, cell recognition, and cell adhesion [37]. The envelope and gag proteins were both subjected to transmembrane helices detection with the aid of the PSIPRED server. The envelope protein elicited four TMH with a score (5.06755), while gag protein showed one TMH with a relatively small score (0.112). Also, the envelope and gag structural proteins of the virus were determined to have a higher antigenic score using the VaxiJen v2.0 (0.4). Accordingly, the envelope and gag proteins were selected for epitopes prediction in this study.

Challenges are undertaken to design a vaccine that takes various factors into account, with safety and effectiveness being paramount [38]. The toxicity and allergenicity of the vaccine-predicted epitopes were considered to ensure the safety of the epitopes [39, 40]. The immunogenicity of the vaccine, the solvent accessibility of amino acids, and the recognition of B cells and the MHC-1 molecules were also considered to ensure the effectiveness of the epitopes [40–42].

In this study, 100% conserved epitopes sequences from envelope and gag proteins were subjected to the ABCpred server. The resultant B cells epitopes were undergone antigenicity, allergenicity, and toxicity analysis. These epitopes were then sorted according to their antigenicity scores starting from the highest score (Table 1). The first eight B cells epitopes which were antigenic, non-allergic, and nontoxic were chosen to enter the assembly of the vaccine construct. The IEDB MHC-1 binding prediction tools were used to identify the specific T cell epitopes that interacted against MHC-I alleles for each of the reference sequences of the envelope and gag proteins. The first eight MHC-1 epitopes from both the envelope and gag proteins demonstrated high binding affinity to MHC-1 alleles, got the highest antigenicity score by the VaxiJen server, and showed no allergenicity or toxicity effects hence were picked to enter the vaccine construct.

To assemble the vaccine construct, the 16 elected B cells epitopes, as well as, the 16 elected T cells epitopes were fused with the aid of suitable protein spacers or linkers. Linkers have displayed an increased significance in the assembly of stable, bioactive fusion proteins [43]. The direct fusion of functional domains without a linker may end with many adverse outcomes such as protein misfolding [44], decreased rate of protein production [45], or diminished bioactivity [46]. In this study, the linkers GPGPG were used to fuse the B cells epitopes, while KK linkers were combined with the T cells. This linking allowed the creation of a sequence with minimal junctional immunogenicity [47]. Additionally; the EAAAK linker at the N-terminal of the vaccine construct was used to add the β-defensin-TLR-3 agonist as an adjuvant to improve the vaccine's immunogenicity. EAAAK are helical linkers used to control the distance and decrease the interference between the domains at a high level of expression [48, 49]. While the β-defensin was used as an adjuvant since it was relatively small in size (45 amino acids), as well as, its capability to perform as an immunomodulator and antimicrobial agent [50]. Lastly, a 6-H tag was added to the vaccine construct at the carboxyl-terminal not to alter the protein structure, for downstream assays, and purification, in addition, could easily define protein function [51, 52].

Most importantly, the vaccine construct was assessed physio-chemically, immunologically, and structurally via bioinformatics tools to endorse the validity and potency of the construct. By using the Protparam server; the physicochemical properties were assessed and 499 amino acids were contained in the protein structure with a molecular weight of 53,324.64 Dalton. In addition, the vaccine construct instability index (II) rated it as a stable vaccine. Furthermore, since the vaccine construct contained aliphatic side chains, the aliphatic index was 56.51 suggesting a potential hydrophobicity. As a result, the GRAVY was -0.731and characterized the vaccine as hydrophilic. To ascertain the antigenicity and allergenicity of this construct; it was assessed by the VaxiJen and the AllerTOP servers to show up non-allergenicity and antigenicity with a high score was 0.8932. Moreover, the predicted vaccine structure demonstrated no transmembrane helix regions, this could facilitate the expression of the vaccine [53]. Accordingly, the overall physicochemical properties demonstrated the chimeric vaccine as thermally stable and suitable to apply as a vaccine against pulmonary adenocarcinoma.

Structural assessment of the vaccine was carried out by the assessment of the secondary and tertiary structure of the vaccine construct. Based on secondary structure analysis, the construct consisted of α-helices, extended strands, β-sheets, and random coils. The best score of the 3D structure of the vaccine construct was selected and then vastly improved, to create a moderately accurate template-based protein model nearer to the native state through conformational sampling with the aid of the galaxy Web refining software [54, 55]. Overcome one of the main problems faced in structural biology is how to experimentally and theoretically recognize the errors in models of protein structures [56]. For this essence, the ProSA program was utilized for the prediction of the potential structural and modeling errors in the vaccine refined construct via its Z-score which was -5.06. Based on this score, the overall quality of the vaccine model was accepted as a vaccine candidate with no errors [57].

Solubility is a critical protein structural property that has important implications for therapeutics and use in diagnosis. The solubility of many proteins is low and it affects the heterologous overexpression of proteins, formulation of products, and their stability [58]. The solubility of vaccine protein overexpressed in *E. coli* is an important criterion for many biochemical and functional analyses. The protein sol and SOLpro servers were used to calculate the solubility of the vaccine construct. In a comparison with the solubility of *E. coli*, the Protein sol server showed that the vaccine was soluble [59, 60]. The protein solubility was 0.621, compared to 0.45 of the average solubility of *E. coli* in the population. This result was confirmed by the SOLpro server that predicted solubility upon overexpression with the probability of 0.819106.

Disulfide bonds play a crucial role in protein structure stabilization. Strong disruption of these bonds is associated with the loss of protein function, and activity [61]. The stability of proteins is substantially enhanced by naturally occurring disulfide cross-links [61, 62]. To assess the number and the location of disulfide bonds, the DIpro tool server was used. The total number of cysteine residues was 18 residues and the predicted number of bonds was seven bonds. To introduce additional disulfide bonds into a refined model of the vaccine construct; the Disulfide by Design for disulfide engineering was applied. Disulfide engineering is essential for protein folding and stability. The introduction of novel disulfide bonds into protein structures has been used expansively to improve the stability of the protein, modify functional features, and aid in the study of protein dynamics [63–66]. Moreover, by decreasing the number of possible conformations for a given protein, structural disulfide engineering reduces entropy and increases thermostability [49]. All these factors promised the constructed vaccine in this study as a tolerable vaccine against JSRV.

Protein–protein molecular docking is a way to find the proper placing of the vaccine construct’s protein into the preferred binding site of the MHC-1 receptor. Primarily, in a non-covalent manner, the formation of stable complexes with potential efficacy and more specificity would be carried out [67, 68]. This study validated docking binding affinity with a high negative rating score using the attractive binding energy between the MHC-1 antigen and the vaccine construct. Therefore, the formation of this docked complex is proficiently stimulating the potential protective immune response.

The immune simulation was conducted for mimicking the immune responses of the host due to exposure to the vaccine. The injection of the vaccine provided a high level of immunoglobulins production indicating the development of memory B cells. The increased level of antibodies has coincided with elevated levels of T lymphocytes demonstrating active humoral, and adaptive responses. INF-γ was also analyzed and the result showed the responsiveness of immune cells to the production of cytokines due to the vaccine (antigen) exposure. The molecular dynamics simulation provided the least RMSF, indicating areas with high peaks showing the increased flexibility and stability of the vaccine. Thus MDS significantly showed the stability of the vaccine by simulating or mimicking the vaccine in the biological milieu as previously described [69–71].

Indeed, the expression of the constructed vaccine in an appropriate *E. coli* expression vector would determine the production of recombinant protein [57]. The assembled vaccine was reverse transcribed and adapted for *E. coli* strain K12 before cloning into the pET-30a ( +) vector. The index of the codon adaptability was 1.0% besides the GC-content was 52.037% enhancing bacterial expression of the protein. Multiple cloning sites were typically used for the cloning of the vaccine construct gene. Thus, the vaccine could be successfully cloned.

## Conclusion

Ovine pulmonary adenocarcinoma is a serious contagious oncogenic and wasting disease spreading all over the world. Up to date, no vaccine against OPA is obtainable, thus the development of a safe and effective vaccine could reduce or eradicate this infectious disease. This study was dedicated primarily to the construction of a novel polypeptides vaccine from envelope and gag proteins of the JSRV disease. The assessments of the vaccine candidate were based on structural and immunological analysis, as well as, molecular docking and cloning studies. Given that B and T cells epitopes have been selected in the final construct and were shown to stimulate both cellular and humoral immunity. Taken together, the proposed JSRV recombinant protein has the potential to be used as a vaccine agent against ovine pulmonary adenocarcinoma.

## Methods

### JSRV proteins’ sequences retrieval

The entire viral proteome of JSRV was retrieved from the UniProt database [72]. JSRV revealed 5 proteins; their names, accession numbers, length, and the other feature of each protein were shown in (Table 3).

### Proteins antigenicity and transmembrane topology

**Fig. 10:**
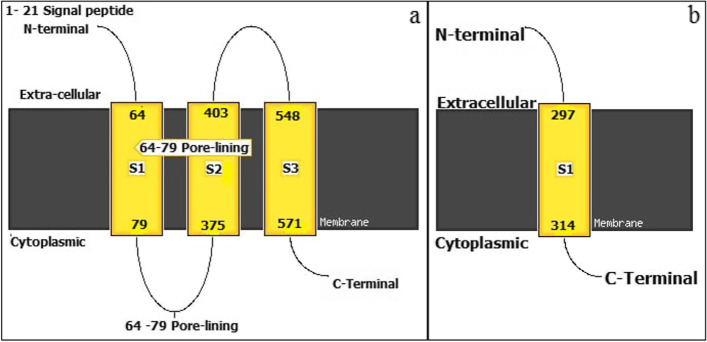
A cartoon of the transmembrane helix topology illustrating the transmembrane helix topology, indicating the proteins' extracellular and intercellular regions. Yellow-filled boxes with labels S1, S2, and S3 represent regions of the transmembrane helix. Membrane regions are denoted by filled-black boxes. An individual line is depicted for the terminal regions N and C. **a** transmembrane helix for the envelope protein (topology: 64–79,375–403,548–571, Helix count: 3, N-terminal: out, Score: 9.0281). **b** transmembrane for the gag protein (topology: 297–314, Helix count: 1, N-terminal: out, Score: 0.112)

The antigenicity of each viral protein was tested to ascertain their potential antigenicity by using the VaxiJen v2.0 server, passing the threshold of (0.4) [73, 74]. The envelope and gag proteins were shown to be antigenic, and they obtained the highest antigenicity score among the five virus proteins (Table 3). The transmembrane topology prediction was tested with the aid of MEMSATSVM Transmembrane helix prediction in the PSIPRED server. This server is a greatly precise predictor of transmembrane helix topology. It is proficient in discriminating signal peptides and finding the cytosolic and extra-cellular loops [75, 76]. Although all the five proteins of the virus demonstrated transmembrane helices, the envelope and gag proteins showed the greatest antigenicity scores with THMs (Fig. 10). Thus the enveloped and gag proteins were appointed as proteins for epitopes prediction in this study.

**Table 3:** Physical and chemical properties, antigenicity, and number of the predicted transmembrane helices of Jaagsiekte sheep retrovirus proteins

**Accession number**	**Protein name**	**MW**	**II**^a^	**AI**	**pI**	**L**	**EC**	**GRAVY**^b^	**VA** ^c^	**TMHs**
***P31621***	Envelope protein	69.34	36.84	99.09	8.57	615	105,770	-0.021	0.6007	Yes/4
***P31622***	Gag protein	68.08	47.03	70.82	7.16	612	78,840	-0.542	0.5465	Yes/1
***P31625***	Neutral protease large subunit	31.31	27.87	98.86	8.9	289	44,920	-0.094	0.4948	Yes /1
***P31623***	Pol protein	88.69	40.83	88.01	5.55	777	115,170	-0.227	0.4876	Yes/1
***P31624***	Hypothetical protein	19.35	38.39	138.49	8.57	166	16,960	0.81	0.4876	Yes/4

*MW* Molecular weight (kilodalton), *AI* Aliphatic index, *pI* Theoretical isoelectric point, *L* Length of the protein, *EC* Extinction coefficient

^a^II: instability index < 40 considered the protein stable

^b^GRAVY negative sign indicated the protein is hydrophilic

^c^Vaxijen antigenicity with a default threshold of 0.4. THMs: Transmembrane helices

### Strains retrieval of envelope and gag proteins

The strains of both envelope and gag proteins were retrieved according to their accession numbers, country, and date of collection from the UniProt database [68]. The retrieved strains of the two proteins were presented in (Table 4).

**Table 4:** The retrieved strains with accession numbers, countries, and year of collection of envelope and gag proteins

Envelope protein			Gag protein		
**Accession**	**Country**	**Year**	**Accession**	**Country**	**Year**
***P31621***^a^	South Africa	1992	***P31622***^a^	South Africa	1992
***A0A0F6UUH9***	China	2013	***A0A482KG78***	India	2017
***A0A1V0CIK3***	China	2013	***Q95N68***	Scotland	2001
***A0A1V0CIK3***	China	2013	***A0A6C0VCI6***	India	2017
***R4V9L7***	China	2010	***P31622***	South Africa	1999
***I4CHA9***	China	2011	***A0A0F6UV80***	China	2013
***Q90RI3***	USA	2004	***P31622***	USA	1992
***Q90RI3***	South Africa	1999	***P31622***	South Africa	1992, 1999,2008

^a^Reference sequence

### Multiple sequence alignment and epitopes conservancy

The retrieved strains sequences of the envelope and gag proteins were aligned and analyzed using multiple sequence alignment (MSA). This was carried out with the aid of the ClustalW in the BioEdit program, version 7.2.5y [77]. MSA of the retrieved strains was exploited to determine 100% conserved epitopes interacted against the B and T lymphocytes. The reference sequences of the envelope and gag proteins were used as an input for each protein analysis.

### Prediction of B cell epitopes

Epitopes on the B-cells play an important role in the development of multi-epitope peptide vaccines and are used in allergy research and disease diagnosis. The reference sequences of envelope and gag proteins were used to predict linear B-cell epitopes, which was performed by the ABCpred server. In this bioinformatics tool, Recurrent Neural Network (RNN) was utilized using a set of non-redundant B-cell epitopes and non-epitopes from the Bcipep and Swiss-Prot databases, respectively [78, 79]. The standard threshold of the server was (0.51) and epitopes length was 12-mer. The resultant predicted epitopes from each protein were evaluated for their conservancy, antigenicity, allergenicity, and toxicity to be selected for the next steps.

### T-lymphocytes epitopes prediction

The IEDB server was used for the identification of the T cell epitopes. Both types of the major histocompatibility complex class I and II can be predicted by this server [80]. Unfortunately, only the ovine or bovine MHC-1 alleles were planned, since the genome project of IEDB has not yet assembled a complete sequence of the ovine or bovine MHC-П locus.

### MHC-I Binding predictions

Based on the IEDB MHC-I prediction tool, peptides from the envelope and gag proteins bound to the bovine MHC class I molecules were analyzed. The prediction method was attained by the Artificial Neural Network (ANN). Before the prediction step, the length of the epitopes was customized as 9mers [80]. Epitopes that bound to alleles with a score equal to or less than one percentile rank were examined for conservancy. The conserved epitopes were further assessed for antigenicity, allergenicity, and toxicity. Based on the results of these assessments the MHC-1 epitopes were picked for additional analysis.

### Epitopes antigenicity, allergenicity, and toxicity analysis

The VaxiJen v2.0 server with a threshold of (0.4), was used to determine the antigenicity of the predicted epitopes [72]. The AllerTOP server was applied to assess the allergenicity [81]. Since the allergen proteins induce an IgE antibody response, the designed vaccine candidate must not exert an allergic impact on the host. The design and prediction of toxic peptides were determined by ToxinPred [82].

### The assemblage of the multi-epitopes vaccine sequence

The sequence of the vaccine was assembled by linking 16 epitopes against B cells and 16 epitopes against T cytotoxic cells. These epitopes were approved passing the criteria in each prediction tool and displayed antigenicity, non-allergenicity, and nontoxicity. To endorse the immunogenicity of the putative vaccine, the Beta-defensin 103 **(**HBD3**),** UniProtKB- P81534 (D103A_HUMAN) was used as an adjuvant [83]. The adjuvant was inserted into the presumed vaccine through the EAAAK linker (Glu-Ala-Ala-Ala-Lys) at the N terminal of the protein. The MHC-1 epitopes were linked via the GPGPG linker, while the B cells epitopes were linked by the KK linker (lys-lys). For purification and identification of this chimeric vaccine, a six His-tag was added to the C terminal of the protein [43, 84]. The construct vaccine was then subjected to a VaxiJen server to test its antigenicity. In addition, the allergenicity was tested by the AllerTOP v2 server.

### Physiochemical properties of the proposed vaccine

ProtParam is a tool that complies with the computation of numerous physical and chemical parameters for a specified protein deposited in Swiss-Prot or TrEMBL or for a user-submitted protein sequence. Various computed parameters encompassing relative molecular mass, theoretical pI, atomic composition, amino acid composition, estimated half-life, extinction coefficient, instability index, aliphatic index, and GRAVY were assessed [85].

### Structural analysis

#### Vaccine secondary structure prediction

Vaccine secondary structure prediction was carried out using the RaptorX server that predicts the secondary structure (SS), the solvent accessibility (ACC), and the disorder regions (DISO) from the vaccine sequence [86, 87]. The SS offered results in two approaches, among them, is the 3-state secondary structure (SS3) that encompassed α-helix (H), β-sheet (E), and coiled regions (C). The ACC model was calculated by the 3-state solvent accessibility technique of the Raptor X server as solvent-exposed (E), medium (M), and buried residues (B). PSIPRED is a simple and accurate secondary structure prediction tool; that was utilized within this study to detect transmembrane topology for the vaccine construct [88, 89]. Moreover, the TMHMM Server v. 2.0 [90] was used to confirm the TMHs obtained by the PSIPRE server in the vaccine construct.

#### Vaccine tertiary structure prediction, refinement, and validation

Vaccine tertiary structure prediction was predicted by the Raptor X server. The server prediction method used neither template information, nor structural characteristics of the protein sequence. Also, the server performed better than other servers, especially for proteins without many homologs in PDBs. The PDB files with the highest Raptor X score were sent to the GalaxyWEB server for structural refinement and quality structure improvement [91]. Galaxy WEB provided five refined PDB models. The model with a high RAMA favored score (92.2) was chosen for Ramachandran plot analysis to validate the protein structure [92, 93]. The potential errors in the vaccine structure were analyzed by using the ProSA server on the refined PDB file obtained by the Galaxy WEB server. The ProSA server displayed a Z-score for experimentally determined protein structures in PDB databases [56].

#### Solubility of the vaccine construct

The Protein sol server is an online prediction algorithm that enables theoretical calculations of protein solubility determination [94, 95]. The refined model of the vaccine construct was submitted to this server, and the data from public databases were used to compare the solubility of the vaccine construct. This server uses the QuerySol (scaled solubility value) to calculate the solubility of a particular protein. In the experimental dataset (PopAvrSol), the average solubility is 0.45, implying that proteins with a solubility of more than 0.45 would be more soluble than the average *E. coli* proteins. The SOLpro tool has been applied to confirm the tendency of the vaccine to be soluble based on the server threshold of ≥ 0.5 is considered to be soluble during over-expression in E. coli [96].

#### Stability of the vaccine construct

Disulfide bonds are a significant key role in protein structure stability, since they form cross-links between the various regions of the polypeptide chains, with disruption strongly accompanying loss of protein function and activity [60]. To detect the number and location of disulfide bonds, the DIpro server was used. DIpro is a cysteine disulfide binding predictor based on a 2D recurrent neural network, support vector machine, graphic matching, and regression algorithms. The server predicts whether the protein sequence has disulfide bindings or not, estimates the total number of disulfide bonds, and predicts both the binding status of the individual cysteine and the linked pairs [97]. Disulfide by Design 2.0 (DbD2) server facilitates disulfide engineering of proteins using a web-based tool. Using DbD2, a refined vaccine construct was designed with disulfide bonds [62].

### Prediction of the 3D structure of the sheep allele

The sequence of sheep allele or ovine MHC class I was retrieved from the UniProt server with UniProt accession number (Q1EMA7) [98]. To predict the 3D structure of the sheep allele; the later retrieved sequence was submitted to the Raptor X server to predict the three-dimensional structure of the allele. The allele’s PDB model with the greatest value was chosen for molecular docking analysis.

### Immunological analysis

#### Molecular docking of the vaccine construct with MHC Class 1 antigen

To study the most favored binding position between the vaccine (ligand) to the analogous receptor, as well as, to have a ligand-receptor complex with the lowest binding free energy molecular docking was performed. The process was applied with the aid of the ClusPro server which is the first entirely computerized tool generally used for protein–protein docking with computational analysis [99, 100]. The PDB file of the refined vaccine was submitted to the server as a ligand and ovine MHC class I antigen (PDB ID: Q1EMA7) as a receptor for the molecular docking. From the 30 best-docked conformations shown on CluPro’s result page, the model with a weighted score of the lowest energy was downloaded in a PDB format. This PDB was then visualized using the PYMOL tool [101] to yield high-quality 3D images analyzing the interaction pattern of the ligand and the receptor.

#### Immune simulation

To analyze the immunogenicity of the vaccine to elicit immune responses, in silico immune simulations were performed using the C-ImmSim server [102]. The vaccine was twice injected within 30 days interval. The Simpson index of diversity was analyzed and interpreted from the simulation plot.

#### Molecular dynamic simulation (MDS)

GROMACS program was used for MDS and energy minimization aiming to elucidate the stability of the vaccine within the biological milieu [103]. The simulation process was conducted as previously described [104] with minor modification. The backbone, potential energy and radius gyration and the RMSF were calculated.

#### Adaptation of codons and in silico cloning

Clonal expression of the refined vaccine constructs in a selected host, E. coli (strain K12), was the goal of this process. Vaccine protein was reverse translated to DNA sequence by JCAT server [105]. The best codon redundancy and the GC content in the DNA sequence are supposed to range between 1.0–0.8 and 30%-70%, respectively. The DNA sequence was modified by adding BamHI and Xho1 restriction enzyme cutting sites at the N- and C-termini, respectively. By using SnapGene software [106, 107], the DNA sequence was inserted into a pET-30a ( +) vector using the BamHI and Xho1 sites. SnapGene proposes the fastest and easiest way for planning, visualizing, and documenting DNA cloning and PCR.

## Supplementary Information

**Additional file 1:** **Supplementary Table 1. **Analysis of the predicted B cell epitopes. The antigenicity, allergenicity, and toxicity of epitopes from the envelope and gag proteins was also assessed. **Supplementary Table 2. **The predicted MHC1 cytotoxic T cells epitopes, their antigenicity, allergenicity, and toxicity of the envelope and gag proteins.

## Data Availability

The datasets generated and/or analyzed during the current study are available in the [UniProt database] repository, [https://www.uniprot.org/uniprot/?query=&sort=score]”. All data, accession numbers/web links are in their final form.

## Abbreviations

3D structure: Three-dimensional structure
ACC: Solvent accessibility
ANN: Artificial Neural Network
B: Buried residues
BAC: Bronchioloalveolar cancer
C: Coiled regions
DISO: Disorder regions
E: Solvent-exposed
E: β-Sheet
Env protein: Envelope protein
GRAVY: Grand Average of Hdropathicity
H: Helix
HBD3: Human Beta-defensin
IC50: Half-maximal inhibitory concentration
IEDB: Immune Epitope Data Base web server
JCAT: Java Codon Adaptation Tool
JSRV: Jaagsiekte Sheep Retrovirus
M: Medium
MDS: Molecular Dynamic Simulation
MHC 1: Major Histocompatibility Complex class I
MSA: Multiple Sequence Alignment
OPA: Ovine Pulmonary Adenocarcinoma
PCR: Polymerase Chain Reaction
PDB file: Protein Data Bank file
pI: Isoelectric point
PopAvrSol: Population Average Solubility
QuerySol: Query Solubility
RefSeq: Reference sequence
RMSF: Root Mean Square Fluctuation
SS: The secondary structure
TM: Transmembrane
TMHMM: Transmembrane helices prediction method based on a hidden Markov Model
TMHs: Transmembrane helices
