# Supplementary material for: A novel strategy for developing vaccine candidate against Jaagsiekte sheep retrovirus from the envelope and gag proteins: an in-silico approach
Source: BMC Vet Res. 2022 Sep 10;18:343. doi: 10.1186/s12917-022-03431-0 (PMC9463060; doi:10.1186/s12917-022-03431-0)
Supplement: Supplementary file 1 — Additional file 1: Supplementary Table 1. Analysis of the predicted B cell epitopes. The antigenicity, allergenicity, and toxicity of epitopes from the envelope and gag proteins was also assessed. Supplementary Table 2. The predicted MHC1 cytotoxic T cells epitopes, their antigenicity, allergenicity, and toxicity of the envelope and gag proteins. [file 12917_2022_3431_MOESM1_ESM.docx]

**Supplementary Table 1:**

Analysis of the predicted B cell epitopes. The antigenicity, allergenicity, and toxicity of epitopes from the envelope and gag proteins was also assessed

| **Protein** | **Epitope** | **Start** | **Prediction**  **Score** | **Vaxijen#**  **antigenicity** | **Allergenicity** | **Toxicity** |
| --- | --- | --- | --- | --- | --- | --- |
| **Envelope protein** | *GTKYGDVGVTGF | 278 | 0.61 | **1.7111** | Non-Allergenic | Non-Toxin |
|  | *HISPQQANISFY | 120 | 0.66 | **1.6667** | Non-Allergenic | Non-Toxin |
|  | *EQVQSINFRMKI | 451 | 0.64 | **1.6045** | Non-Allergenic | Non-Toxin |
|  | *QYPMCFSYQSQH | 136 | 0.62 | **1.402** | Non-Allergenic | Non-Toxin |
|  | *GLTTQYPMCFSY | 132 | 0.6 | **1.3312** | Non-Allergenic | Non-Toxin |
|  | *ISGIDEKTGKKS | 164 | 0.68 | **1.2867** | Non-Allergenic | Non-Toxin |
|  | *HLSIGIGIDTPW | 191 | 0.65 | **1.2565** | Non-Allergenic | Non-Toxin |
|  | *YPRVTISGIDEK | 159 | 0.59 | 1.2294 | Non-Allergenic | Non-Toxin |
|  | TLSLNIYHLNCS | 310 | 0.69 | 1.1754 | Non-allergenic | Non-Toxin |
|  | YRGQHPPIFSVN | 237 | 0.72 | 1.1401 | Non-allergenic | Non-Toxin |
|  | GDVGVTGFLYPR | 282 | 0.7 | 1.1391 | Non-allergenic | Non-Toxin |
|  | SVYNINNANATF | 211 | 0.72 | 1.0540 | Non-Allergenic | Non-Toxin |
|  | PLDIPFCDKHLS | 182 | 0.73 | 0.9552 | Non-Allergenic | Non-Toxin |
|  | MLIQGHMEITLS | 301 | 0.7 | 0.9235 | Non-Allergenic | Non-Toxin |
|  | SLYLQPNISGTK | 269 | 0.63 | 0.9001 | Non-Allergenic | Non-Toxin |
|  | LLLLLQRVQNGA | 68 | 0.6 | 0.8569 | Non-Allergenic | Non-Toxin |
|  | FSVNTAPIYQTE | 245 | 0.76 | 0.7643 | Non-Allergenic | Non-Toxin |
|  | ADISYPRVTISG | 155 | 0.62 | 0.6538 | Non-Allergenic | Non-Toxin |
|  | VSLITLIATAVT | 388 | 0.6 | 0.6024 | Non-Allergenic | Non-Toxin |
|  | RVLGEQVQSINF | 447 | 0.64 | 0.5688 | Non-Allergenic | Non-Toxin |
|  | PMIQSLGWDREI | 91 | 0.82 | 0.4776 | Non-Allergenic | Non-Toxin |
|  | VMLPVEIAEAWY | 347 | 0.7 | 0.4602 | Non-Allergenic | Non-Toxin |
|  | RVQNGAAAAFWA | 74 | 0.84 | 0.4306 | Non-Allergenic | Non-Toxin |
|  | SLTHQMQRMTLS | 19 | 0.71 | 0.4424 | Non-Allergenic | Non-Toxin |
|  | TALELLQRINTA | 361 | 0.54 | 0.4189 | Non-Allergenic | Non-Toxin |
|  |  |  |  |  |  |  |
| **Gag protein** | *TDTQLNFLPGAY | 361 | 0.65 | **1.3888** | Non-Allergenic | Non-Toxin |
|  | *LNFLPGAYAQIS | 365 | 0.73 | **1.2633** | Non-Allergenic | Non-Toxin |
|  | *QEKGALTSKDEL | 165 | 0.84 | **1.1569** | Non-Allergenic | Non-Toxin |
|  | *PPPPPSLKMHPS | 123 | 0.6 | **1.103** | Non-Allergenic | Non-Toxin |
|  | *DWKQTARACLSG | 308 | 0.61 | **1.0534** | Non-Allergenic | Non-Toxin |
|  | *FKQLKELKIACS | 272 | 0.54 | **0.8964** | Non-Allergenic | Non-Toxin |
|  | *NRQQGIQTSYEM | 340 | 0.52 | **0.8518** | Non-Allergenic | Non-Toxin |
|  | *SSTKTEDLSKVR | 389 | 0.65 | **0.824** | Non-Allergenic | Non-Toxin |
|  | PSLKMHPSDNDD | 127 | 0.59 | 0.7029 | Non-Allergenic | Non-Toxin |
|  | ANSACQAALRPY | 440 | 0.82 | 0.7024 | Non-Allergenic | Non-Toxin |
|  | LSSTDEAELDEE | 140 | 0.65 | 0.5965 | Non-Allergenic | Non-Toxin |
|  | TEDLSKVRQGPD | 393 | 0.79 | 0.4904 | Non-Allergenic | Non-Toxin |
|  | LIGEGPYQATDT | 352 | 0.51 | 0.4682 | Non-Allergenic | Non-Toxin |
|  | QLFVHMLSVMLK | 8 | 0.75 | 0.403 | Non-Allergenic | Non-Toxin |

**^#^**The threshold for the Vaxijen antigenicity was 0.4

*Represents the epitopes inter in the structure of the vaccine construct

**Supplementary Table 2:**

The predicted MHC1 cytotoxic T cells epitopes, their antigenicity, allergenicity, and toxicity of the envelope and gag proteins

| **Protein** | **Epitope** | **Alleles** | **Start** | **End** | **Vaxijen# Antigenicity** | **Allergenicity** | **Toxicity** |
| --- | --- | --- | --- | --- | --- | --- | --- |
| **Envelope protein** | *YKWICVTKK | BoLA-T2C | 468 | 476 | **1.7367** | Non-Allergenic | Non-Toxin |
|  | *HISPQQANI | BoLA-T2C | 120 | 128 | **1.5736** | Non-Allergenic | Non-Toxin |
|  | *ISFYGLTTQ | BoLA-T2a | 128 | 136 | **1.1064** | Non-Allergenic | Non-Toxin |
|  | *RGVAKGEQV | BoLA-HD6 | 330 | 338 | **1.0778** | Non-Allergenic | Non-Toxin |
|  | *GVTGFLYPR | BoLA-T2a | 285 | 293 | **0.9625** | Non-Allergenic | Non-Toxin |
|  | *MKIQCHANY | BoLA-D18.4 | 460 | 468 | **0.8693** | Non-Allergenic | Non-Toxin |
|  | *SLLGGKSDI | BoLA-T2C | 111 | 119 | **0.7804** | Non-Allergenic | Non-Toxin |
|  | *HLNCSNCIL | BoLA-T2C | 317 | 325 | **0.6677** | Non-Allergenic | Non-Toxin |
|  | VLGEQVQSI | BoLA-T2C | 448 | 456 | 0.5497 | Non-Allergenic | Non-Toxin |
|  | LSYNVTKVM | BoLA-D18.4 | 417 | 425 | 0.5148 | Non-Allergenic | Non-Toxin |
|  | IENSPKATL | BoLA-T2b | 515 | 523 | 0.4833 | Non-Allergenic | Non-Toxin |
|  | SLAQSIQAA | BoLA-T2C | 403 | 411 | 0.4217 | Non-Allergenic | Non-Toxin |
|  |  |  |  |  |  |  |  |
| **Gag protein** | *TSYEMLIGE | BoLA-T2a | 347 | 355 | **1.537** | Non-Allergenic | Non-Toxin |
|  | *CFKNLTIAL | BoLA-T2C | 179 | 187 | **1.4302** | Non-Allergenic | Non-Toxin |
|  | *RKKGDLSDF | BoLA-D18.4 | 452 | 460 | **1.3402** | Non-Allergenic | Non-Toxin |
|  | *CLDFDNDEL | BoLA-T2C | 88 | 96 | **1.1209** | Non-Allergenic | Non-Toxin |
|  | *GQPGHRAAV | BoLA-D18.4 | 513 | 521 | **1.0927** | Non-Allergenic | Non-Toxin |
|  | *RQAQRLGEV | BoLA-D18.4 | 240 | 248 | **1.0927** | Non-Allergenic | Non-Toxin |
|  | *NSGCFVCGQ | BoLA-T2a | 506 | 514 | **1.0792** | Non-Allergenic | Non-Toxin |
|  | *AMAAALQGK | BoLA-T2a | 476 | 484 | **1.0711** | Non-Allergenic | Non-Toxin |
|  | VQGNPLPPV | BoLA-D18.4 | 555 | 563 | 0.9880 | Non-Allergenic | Non-Toxin |
|  | VNTPNLCPR | BoLA-T2a | 530 | 538 | 0.9859 | Non-Allergenic | Non-Toxin |
|  | MVLAKQLAF | BoLA-HD6 | 429 | 437 | 0.9442 | Non-Allergenic | Non-Toxin |
|  | KQEEDPLHT | BoLA-D18.4 | 104 | 112 | 0.922 | Non-Allergenic | Non-Toxin |
|  | HMLSVMLKH | BoLA-D18.4 | 12 | 20 | 0.89 | Non-Allergenic | Non-Toxin |
|  | SDFSLAFPV | BoLA-JSP.1 | 250 | 258 | 0.8733 | Non-Allergenic | Non-Toxin |
|  | ALTSKDELV | BoLA-HD6 | 169 | 177 | 0.8629 | Non-Allergenic | Non-Toxin |
|  | PSYDPPPPP | BoLA-D18.4 | 117 | 125 | 0.8506 | Non-Allergenic | Non-Toxin |
|  | GIQTSYEML | BoLA-T2b | 344 | 352 | 0.8308 | Non-Allergenic | Non-Toxin |
|  | PPSKHMSPL | BoLA-T2C | 227 | 235 | 0.803 | Non-Allergenic | Non-Toxin |
|  | FQQQARNKK | BoLA-D18.4 | 491 | 499 | 0.8114 | Non-Allergenic | Non-Toxin |
|  | FLAQEKGAL | BoLA-T2b | 162 | 170 | 0.7529 | Non-Allergenic | Non-Toxin |
|  | NSACQAALR | BoLA-T2a | 441 | 449 | 0.7372 | Non-Allergenic | Non-Toxin |
|  | AQEKGALTS | BoLA-D18.4 | 164 | 172 | 0.7317 | Non-Allergenic | Non-Toxin |
|  | LLKQEEDPL | BoLA-T2b | 102 | 110 | 0.7195 | Non-Allergenic | Non-Toxin |
|  | WKQTARACL | BoLA-T2C | 309 | 317 | 0.7102 | Non-Allergenic | Non-Toxin |
|  | STKTEDLSK | BoLA-T2a | 390 | 398 | 0.6834 | Non-Allergenic | Non-Toxin |
|  | QALPPNDWK | BoLA-T2a | 302 | 310 | 0.6728 | Non-Allergenic | Non-Toxin |
|  | KQTARACLS | BoLA-D18.4 | 310 | 318 | 0.6698 | Non-Allergenic | Non-Toxin |
|  | RQQGIQTSY | BoLA-HD6 | 341 | 349 | 0.6574 | Non-Allergenic | Non-Toxin |
|  | PTAPFTIAM | BoLA-JSP.1 | 287 | 295 | 0.5751 | Non-Allergenic | Non-Toxin |
|  | QGIAMAAAL | BoLA-JSP.1 | 473 | 481 | 0.549 | Non-Allergenic | Non-Toxin |
|  | FLPGAYAQI | BoLA-HD6 | 367 | 375 | 0.5148 | Non-Allergenic | Non-Toxin |
|  | TQALPPNDW | BoLA-D18.4 | 301 | 309 | 0.471 | Non-Allergenic | Non-Toxin |
|  | YMQGIAMAA | BoLA-D18.4 | 471 | 479 | 0.4618 | Non-Allergenic | Non-Toxin |
|  | AALRPYRKK | BoLA-T2a | 446 | 454 | 0.4517 | Non-Allergenic | Non-Toxin |
|  | ELKRLGNLL | BoLA-JSP.1 | 95 | 103 | 0.434 | Non-Allergenic | Non-Toxin |
|  | KTDVQGNPL | BoLA-JSP.1 | 552 | 560 | 0.4338 | Non-Allergenic | Non-Toxin |

**^#^** The threshold for the Vaxijen antigenicity was 0.4

*Represents the epitopes entered in the structure of the vaccine construct
